# Reduction of Nitroarenes to Azoxybenzenes by Potassium Borohydride in Water

**DOI:** 10.3390/molecules16053563

**Published:** 2011-04-28

**Authors:** Yufang Liu, Bo Liu, Ailing Guo, Zhenming Dong, Shuo Jin, Yun Lu

**Affiliations:** 1 School of Chemistry and Chemical Engineering, Shanxi University, Taiyuan 030006, China; 2 School of Science, Beijing Jiaotong University, Beijing 100044, China; 3 Department of Chemistry, Southern Illinois University Edwardsville, Edwardsville, IL 62026, USA

**Keywords:** nitroarenes, azoxybenzenes, potassium borohydride, water, PEG-400

## Abstract

The synthesis of the azoxybenzenes by the reduction of nitroarenes with reducing agent potassium borohydride in water was reported for the first time. PEG-400 was used as a phase transfer catalyst and could effectively catalyze the reduction. The electronic effects of substituent groups play an important role in determining the reduction efficiencies. Electron-withdrawing substituents promote the formation of the azoxybenzene products, while electron-releasing groups retard the reductions to various degrees depending on the extent of their electron-donating ability.

## 1. Introduction

**Scheme 1:**
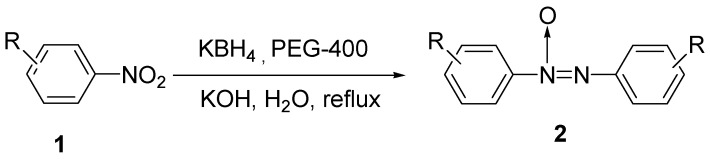
Reduction of nitroarenes to azoxybenzenes.

In 1956, Weill and Panson obtained azoxybenzene from the reduction of nitrobenzene by sodium borohydride in diglyme [1]. Since then, a few investigations on the reduction of nitroarenes by borohydrides were reported [2,3,4,5,6,7]. The reducing agents and the reaction media used include sodium borohydride in dimethyl sulfoxide or sulfolane [2], potassium borohydride in ethanol or pyridine [3], NaBH_4_-BiCl_3_ in methanol [4], NaBH_4_-Bi in ethanol [5,6], and NaBH_4_-(PhTe)_2_ in ethanol [7]. In addition to azoxybenzenes, further reduction products of azobenzenes were sometimes produced depending upon the reaction conditions used. Note that all of these reactions took place in pure organic solvents. Some of them even required pure heavy metals or their compounds as catalyst. This increased both the cost and environmental pollution when using these reactions to synthesize azoxybenzenes. Performing the reduction using a non-toxic borohydride reducing agent in aqueous solution is thus desirable. Herein we reported for the first time the synthesis of the azoxybenzenes by the reduction of nitroarenes by potassium borohydride in aqueous solution (Scheme 1). Poly(ethylene glycol)-400 (PEG-400) was used as a phase transfer catalyst. PEG catalysts have many favorable properties, such as wide availability, low price, nontoxicity, biodegradability, thermal stability, and water solubility. Moreover, their properties can be varied by changing molecular weights. These properties have empowered PEGs as useful phase transfer catalysts in chemical synthesis [8,9,10,11,12]. 

## 2. Results and Discussion

Taking the reduction of *m*-chloronitrobenzene as an example, the effect of the PEG catalysts of various molecular weights on the efficiency of the reaction was investigated. The reactions were performed using 2 mmol nitroarene and 16 mmol potassium borohydride in 20 mL aqueous solution with 2% (w/w) of KOH at reflux temperature conditions. The results are listed in Table 1.

**Scheme 2:**
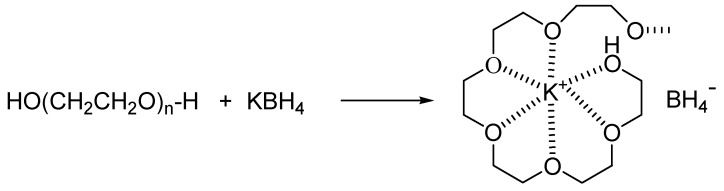
The formation of the PEG-KBH_4_ complex [13,14].

First, we investigated the reduction with or without the presence of the PEG-400 (entries 1-1 and 1-2). The results showed that, with PEG-400, the reaction time was significantly shortened (3 h *vs.* 23 h) and reaction yield was greatly enhanced (85% *vs.* 52%), suggesting that PEG-400 could efficiently catalyze the reduction. This may be because PEG-400 could form a crown ether type complex with the K^+^ in KBH_4_ as shown in Scheme 2, which then brings the BH_4_^-^ reducing species into the organic phase of the oily *m*-chloronitrobenzene (under refluxing conditions, it is in a melted state) for the redox reaction to take place [13,14]. Therefore, the PEG acts as a phase transfer catalyst. Moreover, the effects of various kinds of PEG’s, PEG-600, PEG-800 and PEG-1000 in the reduction reactions were also investigated (entries 1-3 to 1-5). Results showed that increasing molecular weights of PEGs do not affect the reduction efficiency.

**Table 1:** Effects of PEGs on the reduction reaction of *m*-chloronitrobenzene.

Entry	Catalyst ^a^	Reaction time (h)	Yield (%) ^b^
1-1	0	23	52
1-2	PEG-400	3	85
1-3	PEG-600	3	85
1-4	PEG-800	3	85
1-5	PEG-1000	3	85

^a^ The amount of the catalyst is 1 mmol; ^b^ Isolated yields of *m*,*m*’-dichloroazoxybenzene after recrystallization in ethanol.

The reductions of several nitroarenes **1a-l** by potassium borohydride were studied in water catalyzed by PEG-400 at reflux temperature, and the corresponding azoxybenzenes **2a**-**i** were produced in good yields, but the azoxybenzenes **2j**-**l** were not obtained (Table 2). The results suggested that the electronic effects of substituent groups play an important role in determining the reduction efficiencies. It appears that electron-withdrawing substituents promote the formation of the azoxybenzene products, while the electron-releasing substituents retard the reduction to various degrees, depending upon their electron-donating ability. In addition, we found that the reactions of *o*-chloronitrobenzene, *o*-bromo-nitrobenzene and *o*-iodonitrobenzene gave very complicated mixtures which are difficult to separate (not listed in Table 2, detailed studies are under way).

**Table 2:** Reductions of nitroarenes into the corresponding azoxybenzenes by potassium borohydride in water catalyzed by PEG-400 at refluxing temperature ^a^.

Entry	R	Nitroarenes	m. p. (°C) ^b^	Reaction time (h)	Products	Yield (%) ^c^
2-1	*m*-Cl	**1a**	43–45	3	**2a**	85
2-2	*p*-Cl	**1b**	81–83	5	**2b**	78
2-3	*m*-Br	**1c**	56–58	2	**2c**	84
2-4	*m*-I	**1d**	36–38	3	**2d**	80
2-5	*p*-Br	**1e**	125–127	10	**2e**	14
2-6	*p*-Br	**1e**	125–127	4	**2e**	79 ^d^
2-7	*p*-I	**1f**	171–173	10	**2f**	16
2-8	*p*-I	**1f**	171–173	4	**2f**	76 ^d^
2-9	*p*-COOH	**1g**	239–241	2	**2g**	92
2-10	H	**1h**	5.7	4	**2h**	61
2-11	*m*-CH_3_	**1i**	16	10	**2i**	50
2-12	*o*-CH_3_	**1j**	−9.5	24	**NR**	0
2-13	*p*-CH_3_	**1k**	51–52	24	**NR**	0
2-14	*p*-OCH_3_	**1l**	51–53	24	**NR**	0

^a ^Reaction conditions: nitroarenes (2 mmol), potassium borohydride (16 mmol), PEG-400 (1 mmol) in 20 mL aqueous solution with 2% (w/w) KOH at reflux temperature; ^b ^It refers to the melting points of nitroarenes; ^c ^Isolated yields after recrystallization; ^d^ The used reaction solvent is water-ethanol (10 mL:10 mL).

The water-soluble *p*-nitrobenzoic acid (entry 2-9) could react without the presence of the PEG-400, and the reaction results are almost the same as with the PEG. This is because the reaction system is homogeneous and the phase transfer catalyst is not necessary. *p*-Bromonitrobenzene and *p*-iodo-nitrobenzene, whose melting points are higher than 100 °C, exist in solid state in the reaction system. As expected, the reductions were observed to be much less efficient than the reactions with nitroarenes of melting points lower than 100 °C (entries 2-5 and 2-7). In these cases, the phase transfer catalyst did not work. We thus added ethanol to the aqueous solutions of these reactions (50%:50%, v/v) in order to enhance the solubility of the substrates, and the reductions were observed to take place efficiently (entries 2-6 and 2-8).

## 3. Experimental

### 3.1. General

All the reagents were obtained from commercial sources and used without further purification. Melting points of the compounds were measured using a Sanyo-Gallenksmp apparatus and were uncorrected. The IR spectra were recorded on a Perkin-Elmer 1700 spectrometer. ^1^H-NMR spectra were recorded on a Bruker DLX 300 MHz spectrophotometer. Elemental analysis was obtained from a VazioE elemental analyzer.

### 3.2. General Synthetic Procedure

Potassium borohydride (16 mmol), PEG-400 (1 mmol) and nitroarenes (2 mmol) were added in turn to 20 mL aqueous solution with 2% (w/w) KOH in a flask equipped with a condenser. The resulting solutions were magnetically stirred at reflux temperature for a certain period of time until the nitroarenes were consumed. The consumption of the nitroarenes was monitored by TLC. Upon cooling, the mixture was poured into a slurry of concentrated hydrochloric acid and ice. The precipitated product was filtered, washed, dried, and recrystallized.

*m,m’-Dichloroazoxybenzene* (**2a**). Recrystallized from ethanol, m,p. 97–98 °C (lit. [15] 96–97 °C); ^1^H- NMR (CDCl_3_): δ 7.36–7.56 (m, 4H), 7.99 (d, *J* = 7.5 Hz, 1H), 8.19 (d, *J* = 8.4 Hz, 1H), 8.25 (s, 1H), 8.30 (s, 1H); IR, υ (KBr disc): 1556, 1470, 1421, 1302, 783 cm^−1^.

*p,p’-Dichloroazoxybenzene* (**2b**). Recrystallized from ethanol, m.p. 154–156 °C (lit. [15] 156–157 °C); ^1^H-NMR (CDCl_3_): δ 7.47–7.53 (m, 4H), 8.19 (d, *J* = 8.7 Hz, 2H), 8.28 (d, *J* = 9 Hz, 2H); IR, υ (KBr disc): 1583, 1481, 1404, 1323, 831 cm^−1^.

*m,m’-Dibromoazoxybenzene* (**2c**). Recrystallized from ethanol, m.p. 111–113 °C (lit. [15] 109–110 °C); ^1^H-NMR (CDCl_3_): δ 7.36–7.45 (m, 2H), 7.56 (d, *J* = 9.9 Hz, 1H), 7.73 (d, *J* = 9.9 Hz, 1H), 8.06 (d, *J* = 0.9 Hz, 1H), 8.09 (d, *J* = 1.2 Hz, 1H), 8.24 (s, 1H), 8.29 (s, 1H); IR, υ (KBr disc):1553, 1472, 1420, 1298, 781 cm^−1^.

*m,m’-Diiodoazoxybenzene* (**2d**). Recrystallized from ethanol, m.p. 120–121 °C (lit. [15] 118–119 °C); ^1^H-NMR (CDCl_3_): δ 7.18–7.27 (m, 2H), 7.71(d, *J* = 7.8 Hz, 1H), 7.89 (d, *J* = 7.8 Hz, 1H), 8.08 (d, *J* = 9 Hz, 1H), 8.25 (d, *J* = 8.1 Hz, 1H), 8.55 (s, 1H), 8.63 (s, 1H); IR, υ (KBr disc):1545, 1470, 1418, 1294, 783 cm^−1^.

*p,p’-Dibromoazoxybenzene* (**2e**). Recrystallized from ethanol, m.p. 171–173 °C (lit. [16] 173–175 °C); ^1^H-NMR (300 MHz,CDCl_3_): δ 7.62–7.69 (m, 4H), 8.11 (d, *J* = 8.7 Hz, 2H), 8.21 (d, *J* = 9 Hz, 2H); IR, υ (KBr disc): 1575, 1465, 1398, 1319, 826 cm^−1^.

*p,p’-Diiodoazoxybenzene* (**2f**). Recrystallized from ethanol, m.p. 205–207 °C (lit. [16] 208–210 °C); ^1^H- NMR (300 MHz, CDCl_3_): δ 7.74–7.81 (m, 4H), 7.87 (d, *J* = 8.4 Hz, 2H), 7.98 (d, *J* = 8.4 Hz, 2H); IR, υ (KBr disc): 1572, 1468, 1393, 1321, 829 cm^−1^.

*p,p’-Dicarboxylazoxybenzene* (**2g**). Recrystallized from DMF, m.p. > 300 °C (lit. [3] 350–353 °C); ^ 1^H- NMR (DMSO): δ 8.01(d, *J* = 7.8 Hz, 2H), 8.10–8.17 (m, 5H), 8.37(d, *J* = 8.1 Hz, 1H), 13.30 (s, 2H); IR, υ (KBr disc):3421, 1690, 1603, 1460, 1423, 1292, 833；Anal. calcd. for C_1__4_H_1__0_N_2_O_5_: C, 58.74; H, 3.52; N,9.79. Found: C,58.49; H,3.57; N,9.68.

*Azoxybenzene* (**2h). **Recrystallized from ethanol, m.p. 35–36 °C (lit. [17] 35 °C); ^1^H-NMR (CDCl_3_): δ 7.40–7.54 (m, 6H), 8.05–8.32 (m, 4H); IR, υ (KBr disc): 1572, 1474, 1425, 1329, 750, 670 cm^−1^.

*m,m’-Dimethylazoxybenzene* (**2i**). Recrystallized from ethanol, m.p. 35–37 °C (lit. [15] 33–35 °C); ^1^H- NMR (CDCl_3_): δ 2.41 (s, 3H), 2.44 (s, 3H), 7.18 (d, *J* = 7.2 Hz, 1H), 7.33–7.38 (m, 3H), 7.96–8.09 (m, 4H); IR, υ (KBr disc): 1603, 1493, 1458, 1414, 1306, 787 cm^−1^.

## 4. Conclusions

In conclusion, several nitroarenes were reduced in water to the corresponding azoxybenzenes in good yields by potassium borohydride in the presence of PEG-400 as a phase transfer catalyst. Electron-withdrawing substituents on the benzene ring facilitate the reductions. The simplicity of experimental procedure, the high efficiency of the reactions, and the utilization of water as solvent are the advantages of the present protocol.
